# Hidden in Plain Sight? Men's Coping Patterns and Psychological Distress Before and During the COVID-19 Pandemic

**DOI:** 10.3389/fpsyt.2021.772942

**Published:** 2022-01-05

**Authors:** Julianne D. Livingston, George J. Youssef, Lauren M. Francis, Christopher J. Greenwood, Craig A. Olsson, Jacqui A. Macdonald

**Affiliations:** ^1^Centre for Social and Early Emotional Development, School of Psychology, Faculty of Health, Deakin University, Geelong, VIC, Australia; ^2^Centre for Adolescent Health, Murdoch Children's Research Institute, Melbourne, VIC, Australia; ^3^Department of Paediatrics, Royal Children's Hospital, University of Melbourne, Parkville, VIC, Australia

**Keywords:** men, coping, stress, anxiety, psychological distress, depression, COVID-19, pandemic

## Abstract

Individuals cope with stress using multiple strategies, yet studies of coping profiles are rare. We draw data from a longitudinal study of Australian men (*n* = 272; 30–37 years), assessed before (T1) and during (T2) a nation-wide COVID-19 lockdown. We aimed to: (1) identify men's multi-strategy coping profiles before and during the pandemic; (2) assess cross-sectional (T1-T1, T2-T2) and prospective (T1-T2) associations between profiles and symptoms of psychological distress (stress, anxiety, depression, and anger); and (3) examine relationships between coping profiles and appraisals of pandemic-related stressors and options for coping. In latent profile analyses of 14 coping strategies, three profiles emerged that were largely consistent across T1 and T2: (1) Relaxed Copers (low use of all strategies), (2) Approach Copers, and (3) Dual Copers (high avoidant and moderate-high approach-oriented strategies). Compared to Relaxed and Approach Copers, men who were Dual Copers had elevated psychological distress cross-sectionally before (T1) and during (T2) the pandemic, but not prospectively. *Post hoc* analyses suggested this was because many men changed coping profiles in the context of the pandemic. Men with stable (T1-T2) or new (T2 only) Dual Coping profiles experienced greater psychological distress and more negative appraisals of pandemic stressors and options for coping. In sum, at the sample level, the composition of men's coping profiles and associations with mental health risk were relatively stable over time and contexts; however, many men appeared to respond to pandemic conditions by changing coping profile groups, with mostly positive mental health outcomes. Of concern were men who adopted more avoidant strategies (e.g., denial, self-distraction, disengagement, substance use, and self-blame) under pandemic conditions. These Dual Coper men also engaged in commonly observable approach-oriented behaviours (e.g., planning, active coping, humour, seeking practical social support) that may mask their vulnerability to mental health risk. Our findings highlight the clinical importance of enquiring about escalating or frequent avoidant coping even in the presence of more active and interactive approach-oriented behaviours.

## Introduction

Coping refers to cognitive and behavioural efforts to manage stressful situations or their implications [(1), p. 223]. Maladaptive coping patterns are linked to vulnerability and maintenance of psychopathology (2, 3). Prior research has predominantly taken variable-centred approaches that examine associations between individual coping strategies and mental health outcomes. Yet individual coping strategies are rarely used in isolation. Individual differences in the use of multiple strategies–referred to as coping repertoires or profiles–may more meaningfully reflect real-world responses to stress and vulnerability to psychopathology (4). Despite this, little is known about coping profiles and their relevance to psychological distress within populations under stress.

In the emerging research on coping profiles and psychopathology, samples predominantly span adolescence and young adulthood [e.g., (5–7)]. The few adult samples either represent specific subgroups [e.g., breast cancer or trauma survivors, low-income parents; (8–10)] or only report profiles extracted from mixed-gender samples [e.g., (11)]. Yet adult roles and gender norms influence individuals' exposure to stressors and their coping responses (12, 13). Here, we draw on rare longitudinal data to examine adult men's coping repertoires before and during the COVID-19 pandemic and their links with common symptoms of psychological distress (stress, anxiety, depression, and anger).

Prior studies identify links between men's use of avoidance, suppression, and denial with emotional dysregulation, aggression, substance use, and elevated mental health risk (12, 14–16). The picture is less clear regarding relationships between psychological distress and approach-oriented strategies that orient men toward stressors, such as planning, positive reframing, and acceptance. For these, there is mixed empirical support for the theoretically intuitive assumption that approach strategies are adaptive and associated with lower distress (15, 17, 18). Research focused on men's relative use of approach and avoidant coping strategies within their broader coping repertoires may help further understanding of links between coping and psychological distress.

Pre-pandemic research on coping profiles [e.g., (9–11)], and one niche study of French athletes during the pandemic (19) found more severe distress among individual's whose coping profile reflected higher reliance on avoidant relative to approach-oriented strategies. Similar to studies of individual coping strategies (20–22), there are inconsistent reports of associations between poor mental health and coping profiles differentially characterised by frequent approach-oriented coping strategies [e.g., seeking support vs. more independent problem solving; (19)].

One possible explanation is that gender effects in coping tendencies and associated mental health vulnerabilities lead to varying results across samples with differing ratios of men and women [e.g., (10, 11, 23, 24)]. Socialised responses to stress may be particularly pertinent during COVID-19, when strategies previously common in men's coping tendencies, such as active efforts to change the stressor, distraction, and denial (13), may become less or more accessible or adaptive. Whether men's ways of coping with stressors during the pandemic are similar or different to their pre-pandemic coping repertoires and relations with psychological distress can be investigated only with longitudinal data.

Also relevant are cognitive appraisals of the personal threat, harm, or challenge presented by a stressor and perceived options available for coping (25). In combined gender samples, appraisals of pandemic-related stressors as personally threatening and uncontrollable have been negatively associated with approach-oriented coping strategies and positively associated with avoidant coping and symptoms of psychological distress (19, 20, 22, 26). Men's evaluations of what they can do to manage pandemic-related threats or harms may differ depending on the composition of their coping repertoire, particularly their relative reliance on avoidant coping (12), although this has yet to be tested.

Using data from a longitudinal study of men before and during Australia's first wave of COVID-19 infections, we aimed to examine: (1) coping profiles before and during the pandemic; (2) associations between profiles and psychological distress symptoms (stress, anxiety, depression, and anger); and (3) associations between coping profiles and cognitive appraisals of pandemic-related stressors and perceived options for coping.

## Materials and Methods

### Sample

We used data from the Men and Parenting Pathways (MAPP) Study, an ongoing cohort study tracking the health and wellbeing of Australian men (27). Over two years beginning February 2015, MAPP recruited through social media, partner organisations, and word-of-mouth, 608 English-speaking men aged between 28 and 32 years, who were Australian residents, to complete an annual online survey for five years. The MAPP cohort is representative of the geographic spread of socio-economic advantage-disadvantage in Australia, the proportion of men who identify as Indigenous or Torres Strait Islander, and levels of high school education among Australian men of comparable age. MAPP participants are slightly more likely to be in paid employment. For further information about MAPP see Macdonald, Francis (27).

In March 2020, community transmission of the COVID-19 virus was detected in Australia and, by the end of the month, state and territory governments had closed non-essential industries and directed many Australians to stay home unless engaging in essential shopping, caregiving, work or study and limited exercise. The reduced economic activity led to rapid increases in unemployment and financial stress and the delivery of stimulus packages by the Australian Government. By the end of May 2020, community transmission appeared relatively suppressed (28).

In this study, timepoint one (T1) uses data collected from 409 men who completed the third annual MAPP survey between June 2017 and July 2019, prior to the emergence of COVID-19. There was no difference in key demographics of the sample that participated in the third annual survey and the sample at recruitment and the ongoing MAPP cohort (27). Timepoint two (T2) data were collected from 286 ongoing MAPP participants who were invited via email to complete an online survey between 21 March and 19 May 2020 about the impacts of, and their responses to, the COVID-19 pandemic. The T2 sample excluded 14 men who responded to the COVID-19 survey but did not answer coping items (*n* = 272).

MAPP is approved by the Deakin University, Faculty of Health, Human Research Ethics Advisory Group.

### Measures

#### Psychological Distress

At T1 and T2, stress, anxiety, and depressive symptoms were measured using the 21-item Depression Anxiety Stress Scale [DASS-21; (29, 30)]. For each 7-item subscale, participants indicated how frequently they had experienced symptoms during the past week on a 4-point scale from 0 (*Did not apply to me at all*) to 3 (*Applied to me very much or most of the time*). Total subscale scores were doubled for comparison with standardised norms for the 42-item DASS (*normal, mild, moderate, severe, very severe*) [normal, mild, moderate, severe, very severe; (30)]. State anger (i.e., present feelings and urges of anger) was measured because of its association with depression severity in men (16), including in MAPP participants (31). We used the 15-item state anger subscale of the state-trait anger expression inventory (STAXI-2; Spielberger, 1999). Participants rated the intensity of feelings and urges related to anger on a 4-point scale from 0 (*Not at all*) to 3 (*Very much so*). We used standardised population norms (32) to categorise scores as *normal* (0–75^th^ percentile), *high* (75–95^th^ percentile) and *very high* (95–100^th^ percentile) anger intensity.

#### Coping Strategies

At T1 and T2, participants completed the 28-item Brief Cope (33) measuring how often they used 14 strategies (2 items per strategy subscale) to cope with stress on a 4-point scale from 1 (*I haven't been doing this at all)* to 4 (*I've been doing this a lot*). At T2, participants indicated how often they had been using each strategy since the beginning of the pandemic. Approach-oriented strategies were active coping, planning, positive reframing, acceptance, humour, emotional social support, instrumental social support, and venting. Avoidant strategies were denial, self-distraction, behavioural disengagement, substance use, and self-blame. Religious coping (e.g., praying) was not conceptualised as avoidant or approach-oriented, consistent with factor analytic studies in which religious coping failed to load on factors representing either orientation (34, 35).

#### Cognitive Appraisals

At T2, cognitive appraisals of the personal meaning of pandemic stressors were assessed using items adapted from the Cognitive Appraisal Health Scale (36). Participants rated their agreement on a 5-point scale from 1 (*Strongly Disagree*) to 5 (*Strongly Agree*) with 4 items measuring perceived threat (e.g., “*I worry what will happen to me because of COVID-19*”), 4 items measuring perceived harm and loss (e.g., “*I have a sense of loss over things I can no longer do*”), and 3 items measuring perceived ability to overcome challenges to well-being (e.g., “*I can beat the effects of COVID-19 despite the difficulties*”). On the same 5-point scale, participants rated four options for coping with pandemic life effects: (1) could alter something about the situation, (2) had to accept the situation, (3) needed to wait for more information before acting, and (4) had to refrain from preferred way of coping, as per Folkman et al. (37).

#### Potential Confounders

Potential confounders included T1 education level (year 12 or below, trade certificate to advanced diploma, university degree), relationship status, and T1 or T2 subjective financial stress. T1 psychological symptoms were adjusted for in longitudinal and T2 cross-sectional analyses. Confounders used in each analysis are detailed in the Regression Analyses section.

### Analyses

#### Latent Profile Analyses

We used LPA, a person-centred analytic method, to identify classes of men who differ in their patterns of use of 14 coping strategies at T1 and T2. At T1 we first estimated classes using data from all men who participated in the third annual MAPP survey. We then estimated the class solution again at T1 using the subsample of men who later participated in the COVID-19 survey to assess whether classes were consistent and not an artefact of participation bias. LPAs were completed in Mplus version 8.4 (38). Missingness in coping subscales was addressed using full information maximum likelihood during the LPA (39). Two, three, four, and five-class models were estimated at T1. Two and three-class models were estimated at T2 (four and five-class models were not estimable). Model fit was assessed using the Akaike Information Criterion [AIC; (40)], sample-size adjusted Bayesian Information Criterion [aBIC; (41)], Vuong-Lo-Mendell- Rubin Likelihood Ratio Test (VLMR), and the Lo-Mendell-Rubin Adjusted Likelihood Ratio Test [LMR; (42)]. Entropy values indicated class classification accuracy (43). High entropy/classification accuracy (>0.80) enabled us to use participant's coping class membership in the optimal class model at each time point as categorical variables in regression analyses (44).

#### Regression Analyses

Generalised estimating equations were used to assess means and changes in psychological distress variables across T1 and T2. Multiple linear regression (MLR) analyses were used to examine whether coping profiles predicted concurrent and subsequent symptoms of stress, anxiety, depression, and anger at T1 and T2. For each form of psychological distress, a series of MLRs were estimated both unadjusted and adjusted for covariates. In T1 cross-sectional analyses (T1-T1), potential confounders were relationship status, education level, and financial stress assessed at T1. These confounders plus T1 psychological distress were also used in longitudinal analyses predicting T2 distress from T1 coping profiles (T1-T2). In T2 cross-sectional analyses (T2-T2), potential confounders were T1 psychological distress, relationship status, education level, and T2 financial stress. Finally, we used MLR to examine unadjusted associations between T1 and T2 coping profiles and coping appraisals.

All our primary analytical models (i.e., MLRs) were robust to their underlying assumptions (e.g., influential cases using Cook's d <0.20, heteroskedasticity using residual vs. fitted plot, normality of residuals). Whilst there was some evidence for heteroskedasticity and influential cases in exploratory analyses (see *post hoc* investigations section), the magnitude and direction of effects were not meaningfully altered and were largely attributed to small and unequal cell sizes in these analyses. Given these were exploratory analyses, we report the original results and provide results of sensitivity analyses in Supplementary Materials.

Data preparation and MLR analyses were conducted in Stata version 15.1 (45). Variables used in regressions had between 0.4 and 9% missing data that were imputed using multivariate imputation by chained equations (46). Twenty imputed data sets were derived from 50 burn-in iteration of the complete dataset (including 12 auxiliary variables) and pooled using Rubin's (47) rules to derive parameter estimates. Effect sizes were estimated using Cohen's *d* for between group differences and *d*_*av*_ for within-group differences over time (48). Effects were considered significant if pairwise comparisons of marginal means were significant at *p* < 0.05.

## Results

### Descriptive Statistics

Table 1 presents descriptive statistics for the sample and changes in psychological distress over time. Mean age at T1 was 32 years (range 30-35), and at T2 was 34 years (range 31–37). At T1, most participants had completed post-secondary education, were in paid employment, and in a relationship. At T1 and T2, 25.7 and 23.9% of men reported financial stress, respectively. There were nuanced changes in psychological distress symptoms. At T1, the proportion of participants with *moderate* to *very severe* symptoms of stress were 22.60%, anxiety 25.53%, depression 33.65%, and 29.21% reported *very high* anger. At T2, there were small to medium-sized decreases in mean anxiety and anger scores, and a higher probability that men's symptoms were within *normal* levels (anxiety, +7.1%, *p* = 0.029; anger + 50.6%, *p* < 0.001). Mean stress and depression scores and the probability of individuals reporting *normal* symptoms did not change between timepoints. The proportion of men reporting symptoms at each severity level is presented in Supplementary Figure 1, Pearson correlations are in Supplementary Table 2, Supplementary Materials.

**Table 1:** Sample characteristics.

	**T1**		**T2**		**T1 vs. T2**	
	**% | M**	**95% CI**	**% | M**	**95% CI**	***d_***av***_***	**95% CI**
ATSI	1.47	0.00, 2.91				
In a relationship	83.40	78.79, 88.01				
Education level
≤ High school	16.91	12.44, 21.39				
Trade Cert. -Adv. Diploma	33.82	28.18, 39.47				
University	49.26	43.30, 55.23				
Paid employment	97.06	95.04, 99.08				
Financial stress
Comfortable	24.78	19.41, 30.15	35.29	29.59, 41.00		
Doing alright	50.02	43.83, 56.20	40.81	34.94, 46.68		
Just getting by	18.55	13.76, 23.34	18.75	14.09, 23.41		
Difficult	6.65	3.60, 9.71	5.15	2.51, 7.78		
Stress	12.90	11.72, 14.08	13.49	12.37, 14.61	0.06	−0.06, 0.18
Anxiety	**6.36**	5.47, 7.24	**5.00**	4.15, 5.85	**−0.18**	−0.30, −0.06
Depression	10.53	9.30, 11.76	10.94	9.75, 12.13	0.04	−0.08, 0.16
Anger	**29.51**	28.41, 30.61	**22.87**	21.81, 23.93	**−0.73**	−0.86, −0.60

*M, mean; CI, confidence interval; ATSI, Aboriginal or Torres Strait Islander; Cert., certificate; Adv., Advanced; d_av_, Cohen's d for paired data. Estimates derived from pooled values from 20 imputed datasets. Empty cells represent time points when data was not collected. Bold values are significant at p < 0.05*.

### Aim 1: Latent Coping Class Solution

**Figure 1:**
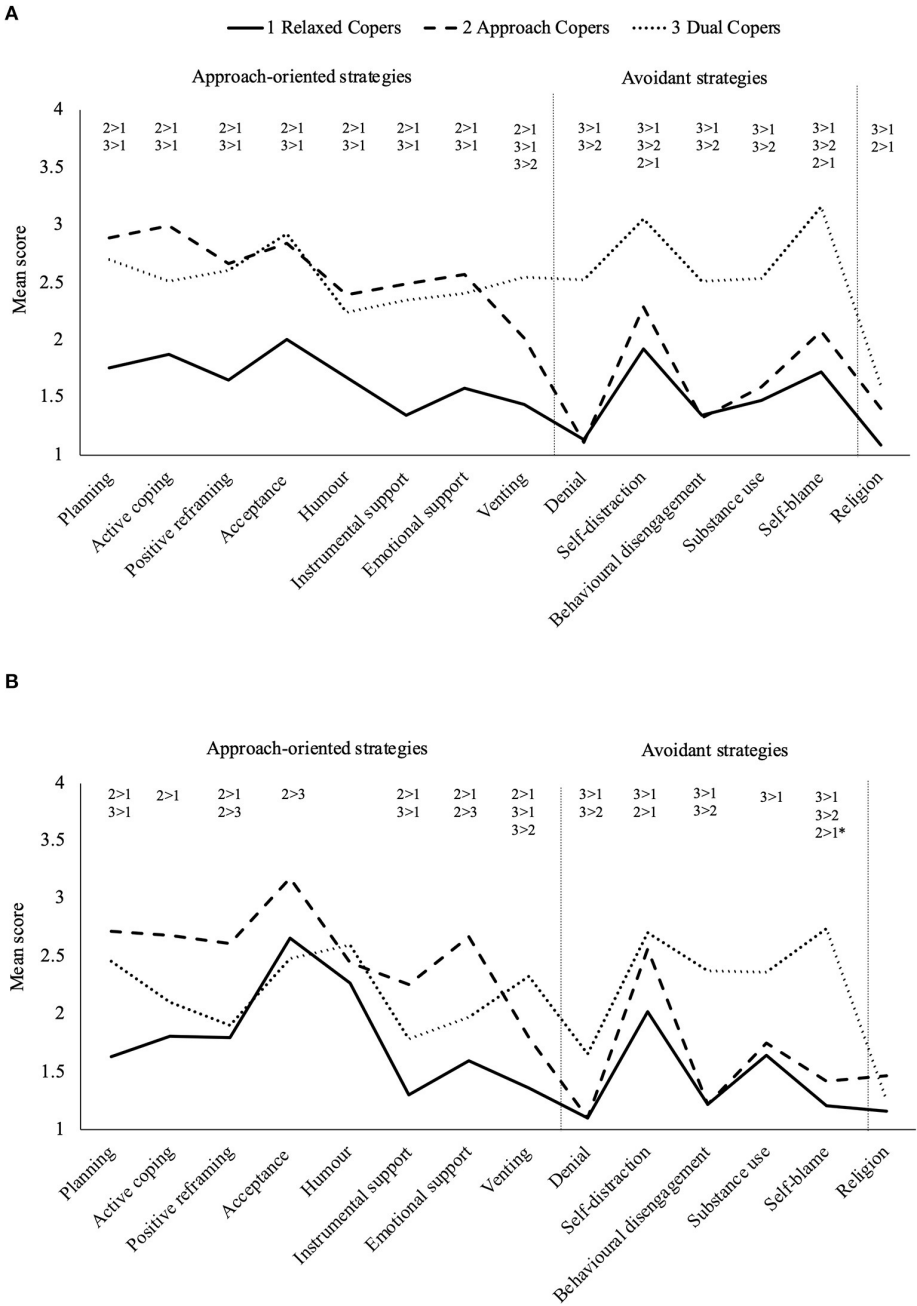
Latent coping classes used by men **(A)** before, then **(B)** during the COVID-19 pandemic. Coping scale range: 1 = *not at all* to 4 = *a lot*. * Near significant contrast with 0.01 95% CI overlap.

LPA fit statistics are presented in Table 2. A three-class model was repeatedly the best fit for coping data from (1) the full sample at T1 (*n* = 409); (2) the subsample at T1 who later participated at T2 (*n* = 260, MI imputed *n* = 272); and (3) the participating sample at T2 (*n* = 272). The three-class model of the T1 subsample had lower AIC and aBIC values and higher entropy (i.e., classification accuracy) than the two-class model. While not significantly better than two classes, visual inspection of coping patterns characterising each class in the three-class model (Figure 1A) and a cross-tabulation of class membership with the optimal 3-class model for the full T1 sample showed very high consistency in class characteristics and membership. Similarly, despite non-significant improvement in fit over the two-class solution, the three-class model at T2 also had lower AIC and aBIC values and stronger classification accuracy than the two-class model and similar characteristics to the three-class models at T1 (Figure 1B). We therefore retained the three-class models for subsequent analyses.

**Table 2:** Model fit indices for LPAs: 2- to 5-class solutions.

**Classes**	**Log likelihood**	**AIC**	**BIC**	**aBIC**	**Entropy**	**VLMR**	**LMR**
**T1 full sample (*****n*** **=** **409)**
2 classes	−6257.59	12601.19	12773.78	12637.33	0.818	0.006	0.006
**3 classes**	**−6027.15**	**12170.30**	**12403.09**	**12219.05**	**0.88**	**0.017**	**0.018**
4 classes	−5901.76	11949.51	12242.52	12010.87	0.86	0.144	0.147
5 classes	−5804.16	11784.31	12137.52	11858.28	0.86	0.447	0.449
**T1 subsample (*****n*** **=** **272)**
2 classes	−4006.56	8099.11	8252.22	8115.90	0.84	0.010	0.010
**3 classes**	**−3854.15**	**7824.30**	**8030.82**	**7846.94**	**0.896**	**0.237**	**0.240**
4 classes	−3757.95	7661.91	7921.84	7690.40	0.882	0.183	0.185
5 classes	−3688.18	7552.36	7865.70	7586.71	0.904	0.246	0.248
T2 sample^*^ **(*****n*** **=** **272)**
2 classes	−3980.19	8046.38	8201.43	8065.09	0.778	0.110	0.112
**3 classes**	**−3834.95**	**7785.90**	**7995.04**	**7811.14**	**0.849**	**0.343**	**0.347**
4 classes	Not estimable					
5 classes	Not estimable					

* *Same as T1 subsample*.

*Bold values indicate retained models*.

### Class Characteristics

The three coping classes exhibited distinct coping patterns across T1 and T2 (Figure 1). Class one reported relatively low and balanced use of all strategies, relying most on acceptance, and self-distraction and were labelled *Relaxed Copers* (T1 *n* = 111, 40.9%; T2 *n* = 150, 55.2%). Class two used more active approach-oriented strategies namely planning, active coping, and acceptance so were labelled *Approach Copers* (T1 *n* = 128, 47.1%; T2 *n* = 86, 31.6%). Class three frequently used avoidant strategies (most often self-distraction and self-blame) and moderate to high use of approach-oriented strategies (including planning and acceptance), so were labelled *Dual Copers* (T1 *n* = 33, 12.1%; T2 *n* = 36, 13.2%). Coping subscale scores and contrasts between classes are presented in Supplementary Tables 3, 4, Supplementary Materials.

While the distinguishing characteristics of the coping classes (i.e., the overall pattern of approach vs. avoidant strategies) were qualitatively stable across timepoints, there were some minor changes in strategy use from T1 to T2. Based on the 95% confidence intervals around mean strategy use at T1 and T2, Relaxed Copers used more acceptance and humour and less self-blame at T2. Approach Copers used less self-blame and Dual Copers reported less positive reframing and denial.

### Aim 2: Associations Between Coping Profiles and Psychological Distress

Table 3 presents adjusted means and standardised differences in symptoms of distress between coping profiles, estimated using MLR models. Dual Copers reported substantially higher concurrent symptoms of stress, anxiety, depression, and anger than Relaxed Copers (*d* = 0.91–1.87) and Approach Copers (*d* = 0.85–1.77) both before (T1-T1) and during (T2-T2) the COVID-19 pandemic. Effects were large, even after adjusting for potential confounders. Before and during COVID-19, Relaxed and Approach Coper's symptoms of stress, anxiety and depression were almost entirely within *normal* levels and their anger on average was *high*. Dual Coper's stress and depressive symptoms were *moderate* to *severe* and their anger *very high*.

**Table 3:** Adjusted means and comparisons of psychological distress and coping appraisals by coping classes at T1 and T2 and longitudinally.

**Outcome**	**Relaxed copers**		**Approach copers**		**Dual copers**		**Approach vs. Relaxed**		**Dual vs. Relaxed**		**Dual vs. Approach**	
	**M**	**95% CI**	**M**	**95% CI**	**M**	**95% CI**	***d***	**95% CI**	***d***	**95% CI**	***d***	**95% CI**
**Stress**												
T1–T1	10.96	9.24, 12.67	12.56	10.92, 14.21	20.76	17.37, 24.14	0.17	−0.08, 0.43	**1.05**	0.64, 1.45	**0.85**	0.46, 1.25
T2–T2	11.88	10.71, 13.06	12.65	11.10, 14.19	22.15	19.59, 24.72	0.10	−0.16, 0.37	**1.37**	0.98, 1.76	**1.27**	0.85, 1.69
T1–T2	13.27	11.70, 14.85	13.92	12.46, 15.37	12.53	9.30, 15.76	0.08	−0.18, 0.33	−0.08	−0.47, 0.30	−0.16	−0.54, 0.22
**Anxiety**												
T1–T1	4.81	3.60, 6.03	5.50	4.39, 6.60	14.93	12.44, 17.41	0.11	−0.15, 0.36	**1.50**	1.08, 1.93	**1.44**	1.02, 1.85
T2–T2	4.08	3.23, 4.93	4.35	3.23, 5.47	10.41	8.55, 12.26	0.05	−0.21, 0.32	**1.18**	0.79, 1.56	**1.12**	0.70, 1.53
T1–T2	4.84	3.71, 5.97	4.87	3.83, 5.90	6.07	3.73, 8.42	0.00	−0.25, 0.26	0.20	−0.19, 0.59	0.20	−0.19, 0.58
**Depression**												
T1–T1	10.12	8.38, 11.87	8.75	7.11, 10.39	18.88	15.42, 22.35	−0.15	−0.40, 0.11	**0.91**	0.51, 1.32	**1.05**	0.65, 1.45
T2–T2	9.19	8.01, 10.36	10.65	9.08, 12.21	18.96	16.36, 21.56	0.20	−0.07, 0.46	**1.31**	0.92, 1.70	**1.10**	0.68, 1.51
T1–T2	10.76	9.19, 12.33	11.23	9.75, 12.70	10.45	7.28, 13.63	0.06	−0.20, 0.31	−0.04	−0.42, 0.35	−0.09	−0.47, 0.29
**Anger**												
T1–T1	27.49	25.79, 29.20	29.08	27.52, 30.64	38.01	34.68, 41.35	−0.08	−0.08, 0.43	**1.13**	0.72, 1.54	**0.97**	0.58, 1.37
T2–T2	21.13	20.11, 22.15	21.59	20.23, 22.95	33.19	30.96, 35.42	−0.19	−0.19, 0.34	**1.87**	1.45, 2.27	**1.77**	1.32, 2.22
T1–T2	22.94	21.48, 24.40	22.28	20.95, 23.61	24.97	21.97, 27.96	−0.34	−0.34, 0.17	0.25	−0.14, 0.64	0.34	−0.04, 0.72
**Appraisals of pandemic stressors**												
Threat	2.39	2.25, 2.52	2.70	2.52, 2.88	2.99	2.71, 3.27	**0.37**	0.11, 0.64	**0.73**	0.36, 1.10	0.34	−0.05, 0.73
Harm	2.59	2.44, 2.74	2.81	2.61, 3.00	3.38	3.08, 3.68	0.24	−0.03, 0.50	**0.85**	0.47, 1.22	**0.63**	0.23, 1.03
Challenge	3.05	2.92, 3.17	3.36	3.19, 3.53	2.56	2.29, 2.82	**0.40**	0.13, 0.70	**−0.65**	−1.01, −0.28	**−1.00**	−1.40, −0.59
**Appraisals of coping options**												
Alter	2.47	2.30, 2.65	3.07	2.84, 3.30	2.28	1.92, 2.63	**0.54**	0.30, 0.81	−0.17	−0.53, 0.19	**−0.73**	−1.13, −0.33
Accept	4.11	3.98, 4.24	4.42	4.24, 4.59	4.03	3.76, 4.30	**0.38**	0.12, 0.65	−0.10	−0.46, 0.27	**−0.48**	−0.87, −0.09
Info	2.87	2.71, 3.02	2.98	2.77, 3.18	3.53	3.21, 3.85	0.12	−0.15, 0.38	**0.70**	0.33, 1.07	**0.58**	0.18, 0.97
Refrain	3.03	2.86, 3.20	3.33	3.10, 3.55	3.69	3.35, 4.04	**0.28**	0.02, 0.55	**0.62**	0.25, 0.99	0.34	−0.05, 0.73

*T1-T1, coping and distress before the pandemic; T2-T2, coping and distress during the pandemic; T1-T2, T2 distress regressed on T1 coping profile. Psychological distress models adjusted for potential confounders. Appraisals regressed on T2 coping profiles. Bold values are significant at p < 0.05*.

In longitudinal analyses (T1-T2), Dual Copers at T1 also had higher subsequent symptoms of stress, anxiety, depression, and anger during the pandemic (T2) than men classified as Relaxed or Approach Copers, but these effects became non-significant after adjusting for T1 symptoms and other potential confounders. There were no differences between Relaxed and Approach Copers in concurrent or future psychological distress symptoms. For unadjusted effects see Supplementary Table 6, Supplementary Materials.

### Aim 3: Associations Between Coping Profiles and Cognitive Appraisals of Pandemic-Related Stressors

Marginal means and standardised differences in coping appraisals between coping profiles are presented in Table 3. Dual Copers at T2 appraised the effects of the pandemic as more personally threatening than Relaxed Copers, and more harmful and difficult to overcome, and more strongly needed more information before acting, than both Relaxed and Approach Copers (*d* = 0.63–1.00). Both Dual Copers and Approach Copers perceived a stronger need than Relaxed Copers to refrain from their preferred way of coping with the effects of the pandemic. In contrast, Approach Copers at T2 judged the personal effects of the pandemic as more threatening than Relaxed Copers (*d* = 0.37), but more strongly perceived these effects as a challenge they could overcome and change directly, compared to Relaxed and Dual Copers (*d* = 0.40–1.00). While all coping classes reported high acceptance of some pandemic impacts, Approach Copers reported higher acceptance than Relaxed and Dual Copers. See Table 3 for all effects. There were no differences between T1 coping classes in cognitive appraisals during the pandemic (results in Supplementary Table 5, Supplementary Materials).

### *Post hoc* Investigations

**Figure 2:**
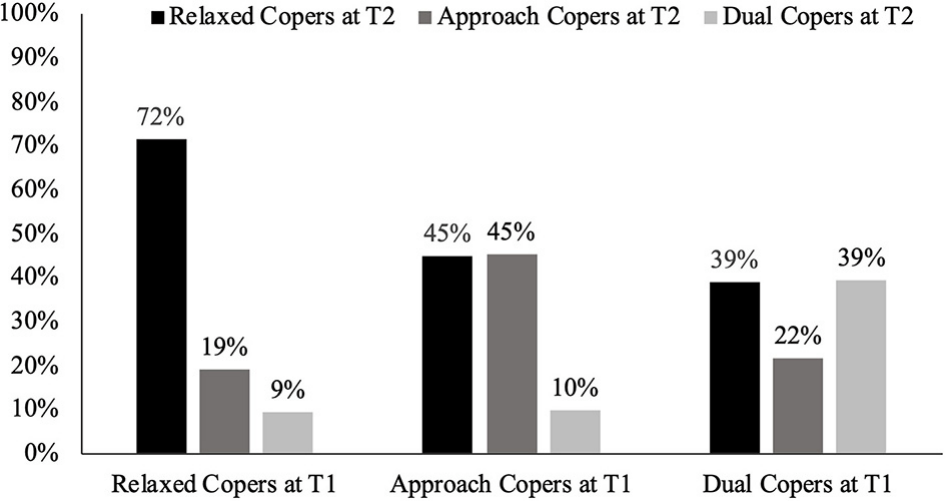
Percentage of each pre-pandemic (T1) coping class classified as relaxed, approach, or dual copers at T2.

We examined whether the stability of coping profiles may help explain the negligible prospective association, after adjustment, between T1 coping profiles and T2 psychological distress symptoms and coping appraisals during the pandemic. Forty five percent of men changed coping profiles between T1 and T2 with most adopting a relaxed or approach-oriented profile, shown in Figure 2.

Given Dual Coping was associated cross-sectionally with higher psychological distress before and during the pandemic (Table 3), we questioned whether men with a stable Dual Coping profile faced a higher mental health risk than men who only adopted the Dual Coping profile during the pandemic. We also queried whether changes in coping profiles were linked to cognitive appraisals of pandemic-related stressors. To answer these questions, we explored whether patterns in profile stability or change was associated with appraisals and psychological distress. To reduce complexity, we combined the Relaxed and Approach Copers classes given their similar relationships with symptoms of distress and member overlap at T1 and T2. This created four longitudinal coping patterns: (1) *Stable Relaxed/Approach Copers* (79.4% of sample) were Relaxed or Approach Copers at T1 and T2; (2) *New Relaxed/Approach Copers* (7.3%) were Dual Copers at T1 but Relaxed or Approach Copers at T2; (3) S*table Dual Copers* (4.8%) were Dual Copers at T1 and T2; and (4) *New Dual Copers* (8.5%) were Relaxed or Approach Copers at T1 but Dual Copers at T2.

**Figure 3:**
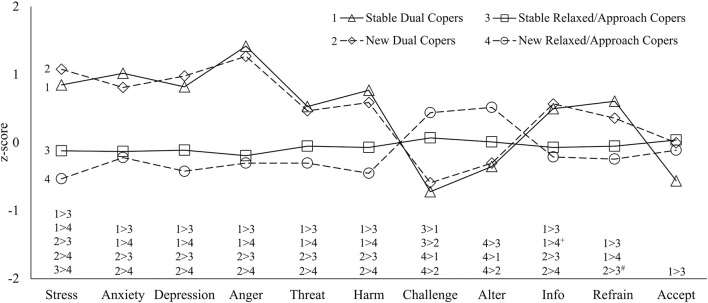
Standardised adjusted mean symptoms of psychological distress and coping appraisals of longitudinal coping patterns. Alter, can alter situation; Delay, need to delay acting until better informed; Refrain, refrain from preferred coping; and Accept, must accept situation. Variables were standardised prior to analyses for figure only. Contrasts are significant at *p* < 0.05, with two exceptions: ^+^*p* = 0.05. ^#^*p* = 0.06.

We substituted these four longitudinal coping patterns for the T1 coping profiles in the unadjusted MLR models used to predict coping appraisals, and in the adjusted and unadjusted longitudinal MLR models used to predict symptoms of stress, anxiety, depression, and anger during the pandemic. Results are presented in Figure 3, with means and effects for all outcomes reported in Supplementary Table 7, Supplementary Materials.

There were no differences between Stable and New Dual Copers in their coping appraisals or adjusted symptoms of distress during the pandemic. Both Stable and New Dual Copers appraised the effects of the pandemic as more personally threatening (*d* = 0.50–0.76), harmful (*d* = 0.69–1.10), harder to overcome (*d* = 0.64–1.14), perceived a greater need to delay acting until more informed (*d* = 0.58–0.76), and experienced substantially higher adjusted stress (*d* = 1.23–1.93), anxiety (*d* = 1.15–1.40), depression (*d* = 1.18–1.75), and anger (*d* = 1.86–2.06) than Stable and New Relaxed/Approach Copers, with mostly large effects. Stable (but not New) Dual Copers reported a stronger need than Stable or New Relaxed/Approach Copers to refrain from their preferred ways of coping (*d* = 0.68–0.76).

In contrast, New Relaxed/Approach Copers more strongly believed they could change their stressful situation (*d* = 0.51–0.84) and experienced lower adjusted stress during the pandemic than other groups (*d* = −0.52-−1.93). While acceptance was high across groups, Stable Relaxed/Approach Copers were marginally more accepting than Stable Dual Copers (*d* = 0.56, *p* = 0.06). For adjusted and unadjusted effects see Supplementary Table 8, Supplementary Materials.

## Discussion

We identified three distinct profiles of coping strategies and their associations with symptoms of psychological distress and coping appraisals among Australian men before and during the COVID-19 pandemic. We found that men who frequently tried to avoid or distract from stressors or their implications had elevated symptoms of stress, anxiety, depression, and anger even though these men also regularly used active, approach-oriented coping strategies (i.e., Dual Coping profile) often considered beneficial for adjustment and mental health (2). In comparison, men who infrequently used any coping strategies except acceptance and self-distraction (i.e., Relaxed Coping profile) or who relied on more approach-oriented strategies (e.g., planning, active coping, acceptance) had lower psychological distress. Effect sizes for cross-sectional associations between coping and distress symptoms were moderate to large before and during the pandemic.

Surprisingly, men's pre-pandemic coping profiles failed to predict subsequent symptoms of psychological distress (after adjusting for potential confounders) or coping appraisals during the pandemic. Our findings suggest this may be because almost half the men coped with pandemic stressors differently to their pre-pandemic coping tendencies. Most men who changed profiles relaxed their overall coping efforts, or, less commonly, increased their use of approach-oriented strategies. These coping patterns were associated with lower psychological distress, lower perceived threat and harm, and stronger belief in their ability to overcome the personal impacts of the pandemic. In contrast, men who increased their use of avoidant coping (while still using approach coping strategies i.e., New Dual Copers), perceived the effects of the pandemic as more threatening, harmful, and difficult to overcome. They also perceived fewer options for coping, and experienced similar levels of distress as men who consistently employed high levels of avoidant coping (i.e., Stable Dual Copers), even after adjusting for baseline distress. These findings provide novel evidence of heterogeneity and flexibility in men's coping patterns and a coping profile that may be a covert indicator of mental health risk with important clinical implications.

Our finding of structurally similar coping profiles across time and contexts suggests these coping strategies converge in predictable ways among men aged in their 30's. While the characteristics of the coping profiles are consistent with some (but not all) past studies using combined samples of men and women [e.g., (10, 11)], we provide novel evidence that almost one in two men changed their coping profile over time and stress-contexts. Past research suggests individuals tailor their coping to the demands of a situation but show stable tendencies in their use of specific strategies over time (49, 50). However, this study extends findings from recent studies of women with breast cancer (8) and mixed-gender samples of Norwegian workers and French athletes (11, 51) to show that almost half of our community sample of men changed their total *and* relative use of multiple strategies across contexts. This led to a shift in men's broader coping repertoire that was also linked with distinct coping appraisals. For most of our sample, this flexibility was associated with positive mental health outcomes. Indeed, men who reduced their previous high avoidant coping when dealing with pandemic stressors most strongly believed they could improve their situation and experienced lower symptoms of stress, adjusted for pre-pandemic levels, than other coping groups [consistent with cross-sectional and longitudinal associations between controllability appraisals and stress, anxiety, and depression *during* the pandemic; (19, 26)].

We also found a minority of men *increased* their avoidant coping during the pandemic and reported coping appraisals and increased psychological distress on par with longer-term Stable Dual Copers. These findings align with stress and coping theory (25) and COVID-19 studies on risk and resilience factors (20, 22, 26). While avoidance can be adaptive (52), inflexible or excess use of strategies typically considered maladaptive may interfere with successful use of approach-oriented coping or impair flexible responding (4, 53). For example, denial may delay time-sensitive action (54) while avoidant and disengagement strategies may reduce sensitivity to environmental feedback and hamper flexible responding (4). Moreover, avoidance and distraction are associated with habitual suppression of vulnerable emotions, which is linked with emotional dysregulation and secondary problems in men (12).

These findings indicate that during large scale stressful events, some men experience multiple indicators of vulnerability to mental health problems. Yet their moderate use of active, interactive, and more observable approach-oriented coping strategies may mask their risk. For example, an individual may use humour or seek practical help from others while relying on minimisation or substances to rigidly avoid stress-induced aversive thoughts and emotions. Moreover, some avoidant strategies may be endorsed as traditional masculine-conforming ways of coping with stress and distress (55). For example, qualitative researchers found men's disclosures of depressed feelings (including irritability) may be minimised or dismissed by some mental health professionals who perceive men's alcohol use and efforts to cope independently as expressions of traditional masculinity and lower openness to treatment (56). In this way, the Dual Coping profile–whether stable or newly adopted–may represent a “masked” or covert risk factor for symptoms of stress, anxiety, depression, and anger among men.

Our finding that men with a (Stable or New) Dual Coping profile most strongly believed they had to delay acting and Stable Dual Copers perceived a stronger need to refrain from their preferred way of coping with the impacts of COVID-19 was partly surprising. In contrast, prior research found a perceived need for more information before acting was associated with higher use of approach-oriented strategies of support seeking and planful problem-solving (37). In our study some men may have had limited access to their social networks during government-mandated lockdowns and felt they had limited control of stressors during the pandemic so turned to avoidance and distraction to manage, reflected in the Dual Coping profile. Moreover, feeling uninformed or receiving misinformation during the initial stages of the pandemic may have fuelled excessive media consumption (57), an approach-oriented strategy that when used inappropriately may constitute a form of ineffective reassurance-seeking previously associated with avoidant coping and anxiety and depressive symptoms during COVID-19 (21). The strong need for refrain and higher anger experienced by Stable Dual Copers has been previously associated with aggression, confrontations with others, and avoidant/escape coping under stress (37, 58–60). While speculative, this suggests long-term Dual Copers may have needed to exercise self-control to refrain from expressing intense (>95^th^ percentile) angry feelings and urges, potentially indicating a risk of verbal or physical aggression.

### Strengths and Limitations

A strength of this study was the longitudinal design which enabled us to test associations between pre-pandemic coping and psychological distress during COVID-19 and explore flexibility in coping patterns and links with cognitive appraisals and mental health risk. Consistent with limited pre-pandemic longitudinal analyses of coping profiles [e.g., (11)], our findings indicate that men's coping profiles may not be highly reliable indicators of future risk, however, trajectories of change in coping profiles may be important indicators of men's vulnerability to mental health problems, if our exploratory findings are replicated. Future research should examine risk and protective factors that predict coping trajectories to inform prevention and intervention for mental health difficulties.

While a fraction of our sample did not provide data at both timepoints, the rate of participation at both timepoints is high compared to other longitudinal studies of men (61) and potential bias was minimised via multiple imputation of missing data (62). Consistent with other cohort studies that investigated the impacts of COVID-19 through comparisons with pre-pandemic data [e.g., (63)], within our sample there were variable time lengths between the collection of pre-pandemic and COVID-19 data. This was unavoidable due to the variation in the timing of participant's annual surveys and the emergence of the COVID-19 pandemic.

While the MAPP cohort is representative of similar-aged men in Australia on key demographics, they reported higher symptoms of stress, anxiety, depression, and anger than population norms (32, 64). This may indicate an elevated risk profile, although evidence suggests prior epidemiological studies of mental health under sampled at-risk men (65). Moreover, evidence suggests online recruitment increases representation of “hard-to-reach” individuals and those with mental health problems (61, 66). Greater sampling of at-risk men may increase our ability to discover relevant relationships between psychological distress and coping. However, our findings may not be generalisable to other subgroups, particularly those differentially impacted by the pandemic [e.g., elderly; (67)]. While we used well-validated self-report measures of psychological distress and coping (29, 68), self-report is vulnerable to bias. Research that triangulates men's self-report with other sources of information such as observant or clinical assessments would strengthen the accuracy and robustness of findings.

### Implications

Our findings have implications for clinicians who work with men and those involved in mental health services generally. We present evidence of three distinct coping patterns used by men and elevated psychological distress among those who frequently engage in avoidant coping, which may be masked by their more visible approach-oriented coping behaviours. Indeed, the Dual Coping profile may be a covert risk indicator that contributes to the under recognition and treatment of men's mental health problems (69). This is important because some men are reluctant or unable to disclose their distress, often delay seeking help until in a crisis (70, 71), and may have their expressions and management of stress and distress overlooked or misunderstood by clinicians influenced by gender biases (56).

Previous research examining men's help-seeking recommends clinicians focus on action-oriented psychological interventions due to men's preference for active, problem-focused strategies and skills (72). However, our findings suggest a simultaneous overreliance on avoidant coping strategies (e.g., denial, distraction, disengagement) may leave some men vulnerable to developing or experiencing ongoing symptoms of distress. This includes feelings and urges relating to anger, which if enacted may result in verbal and/or physical aggression (73), and/or fuel chronic hostility and criticism of self and others that can predispose and perpetuate emotional and relational problems (74, 75).

We also identified cognitive themes that may help identify at-risk men for further assessment and tailored psychosocial support. Consistent with other COVID-19 studies (19), appraisals of pandemic-related stressors as personally threatening, loss-inducing, and uncontrollable were associated with more avoidant coping through the Dual Coping profile. We further found that a perceived need for restraint or delay in responding (until better informed) may be additional indicators of risk, consistent with growing evidence that at-risk men often delay help-seeking (70). These appraisals warrant further investigation given their previous links with interpersonal confrontations, the latter a potential sign of difficulties with aggression, and the exploratory nature of our analyses.

### Conclusions

This study examined links between men's patterns of coping with stress, coping appraisals, and psychological distress over time and contexts, including the first wave of the COVID-19 pandemic in Australia. Men who engaged in high levels of avoidant coping and moderate-high approach-oriented strategies experienced elevated symptoms of stress, anxiety, depression, and anger and cognitive appraisal themes of fear, loss, uncontrollability, delay, and restraint. These findings, if replicated, suggest indicators of men's vulnerability to psychological distress; including risk potentially masked by active and interactive approach-oriented coping typically more visible than some avoidant coping strategies. Most importantly, these findings contribute to ongoing work to identify cognitive and behavioural targets for screening and treatment of men's mental health difficulties.

## Data Availability Statement

The data analysed in this study is subject to the following licences/restrictions: Access to MAPP data is governed by the study investigators and can be initiated via contact with Dr. Jacqui Macdonald (corresponding author). Data sharing must be consistent with ethical approvals for participant consent, confidentiality and data management. MAPP ethics approvals do not include participant consent for public availability of our data sets. However, we support requests for reuse of data for validation, verification or confirmation of previous research, subject to available resources at the time of the request to undertake sufficient deidentification of data for sharing. Requests to access these datasets should be directed to Dr. Jacqui Macdonald, jacqui.macdonald@deakin.edu.au.

## Ethics Statement

The studies involving human participants were reviewed and approved by Deakin University, Faculty of Health, Human Research Ethics Advisory Group. The patients/participants provided their written informed consent to participate in this study.

## Author Contributions

JL, JM, and GY: study conceptualisation and design. JM and CO: longitudinal MAPP study conceptualization, design, and cohort experts. JL, JM, LF, and CG: data collection and preparation. JL overseen by JM and GY: study statistical analyses and interpretation of results. JL overseen by JM: drafting of original manuscript. All authors critically reviewed and approved the final manuscript.

## Funding

JL undertook this research with support from an Australian Government Research Training Program Scholarship. CO was supported by an NHMRC Investigator Grant (APP1175086). JM was supported by a Deakin University mid-career fellowship.

## Conflict of Interest

The authors declare that the research was conducted in the absence of any commercial or financial relationships that could be construed as a potential conflict of interest.

## Publisher's Note

All claims expressed in this article are solely those of the authors and do not necessarily represent those of their affiliated organizations, or those of the publisher, the editors and the reviewers. Any product that may be evaluated in this article, or claim that may be made by its manufacturer, is not guaranteed or endorsed by the publisher.
