# Supplementary material for: Hidden in Plain Sight? Men's Coping Patterns and Psychological Distress Before and During the COVID-19 Pandemic
Source: Front Psychiatry. 2022 Jan 5;12:772942. doi: 10.3389/fpsyt.2021.772942 (PMC8766713; doi:10.3389/fpsyt.2021.772942)
Supplement: Supplementary file 1 [file Table_1.pdf]

Supplementary Materials

Figure 1S. Proportion of Sample in Each Level of Severity of Symptoms of Stress, Anxiety, Depression, and Anger Before (T1, June 2017–July 2019) and During the COVID-19 Pandemic (T2, March–May 2021)

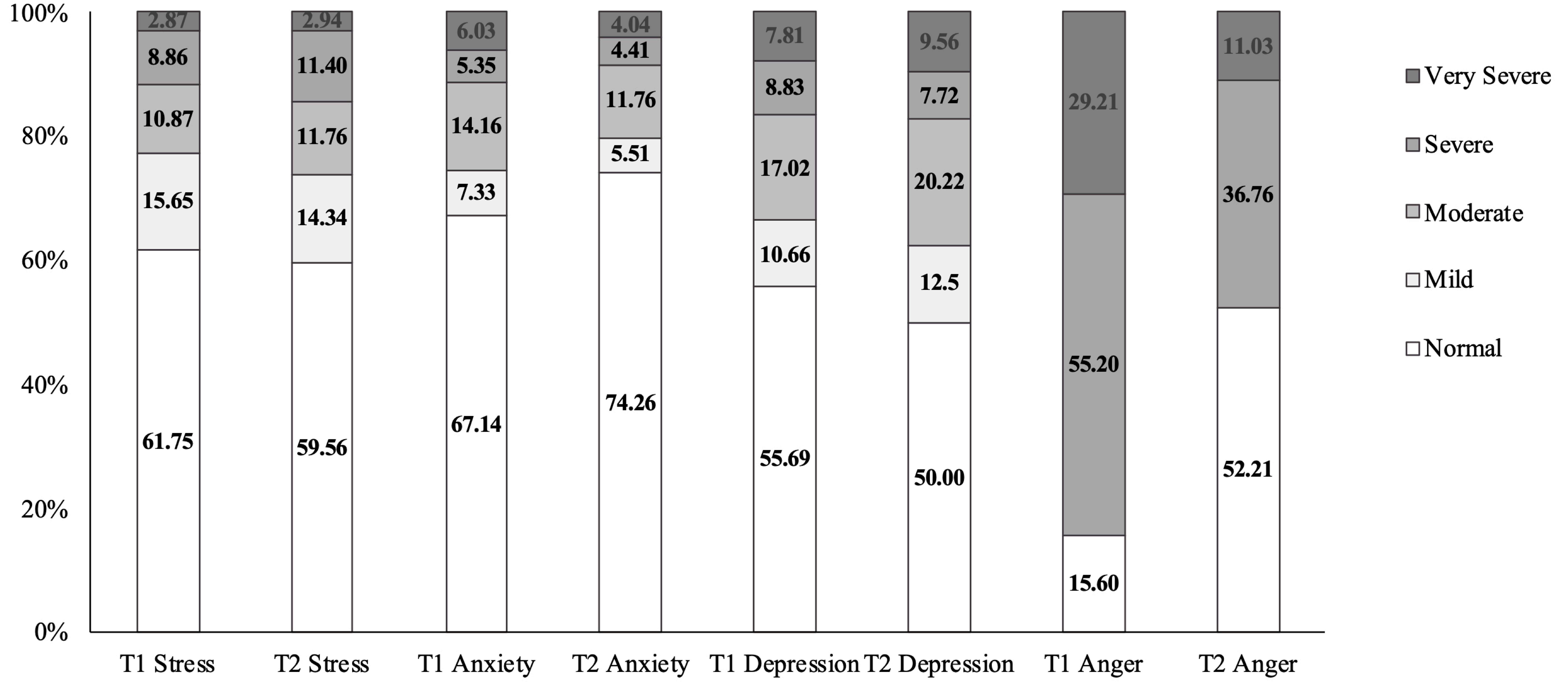

Note. Stress, anxiety, and depression severity categories from DASS-21 manual (Lovibond & Lovibond, 1995). Anger severity category cut-offs for normal and severe represent the 75th and 95th percentile of adult norms for state anger in the STAXI-2 manual (Spielberger, 1999). Anger scores were only separated into three levels of severity consistent with the scale authors' focus. Estimates are pooled values from 20 imputed datasets.
