# Supplementary material for: Hidden in Plain Sight? Men's Coping Patterns and Psychological Distress Before and During the COVID-19 Pandemic
Source: Front Psychiatry. 2022 Jan 5;12:772942. doi: 10.3389/fpsyt.2021.772942 (PMC8766713; doi:10.3389/fpsyt.2021.772942)
Supplement: Supplementary file 2 [file Table_2.pdf]

Table S2. Correlations Between Continuous Variables Used in the Analysis

	Stress T1	Anxiety T1	Depression T1	Anger T1	Stress T2	Anxiety T2	Depression T2	Anger T2	Threat	Harm	Challenge	Alter	Accept	Delay
Psychopathology symptoms														
Stress T1														
Anxiety T1	**.71**													
Depression T1	**.67**	**.62**												
Anger T1	**.62**	**.54**	**.51**											
Stress T2	**.52**	**.37**	**.38**	**.41**										
Anxiety T2	**.40**	**.53**	**.37**	**.34**	**.67**									
Depression T2	**.37**	**.31**	**.56**	**.35**	**.71**	**.61**								
Anger T2	**.40**	**.35**	**.35**	**.43**	**.70**	**.60**	**.62**							
Coping appraisals														
Threat	.13^{+}	.16	**.13**	**.10**	**.28**	**.25**	**.24**	**.22**						
Harm	.11	.15	**.15**	**.16**	**.38**	**.26**	**.38**	**.33**	**.41**					
Challenge	-.12	-.02	-.12	**-.15**	**-.33**	**-.25**	**-.37**	**-.29**	**-.20**	**-.19**				
Alter	-.02	.04	-.06	-.05	**-.20**	-.07	**-.23**	**-.21**	-.10	-.05	**.62**			
Accept	-.03	-.10	.01	-.13^{+}	.02	-.09	.06	.03	.06	.04	.09	-.03		
Delay	.11	**.19**	**.14**	.12^{+}	**.18**	**.22**	**.15**	**.17**	**.29**	**.17**	.01	.10	.09	
Refrain	.02	.09	.06	**.14**	**.17**	**.14**	**.16**	**.19**	**.18**	**.50**	**-.12**	-.08	.08	**.28**

Notes. $N = 272$. T1 = Timepoint 1, before the COVID19 pandemic. T2 = Timepoint 2, during the COVID-19 pandemic. Threat = perceived personal threat of pandemic. Harm = perceived personal harm of pandemic. Challenge = perceived personal challenge of pandemic. Alter = perceived ability to change or do something about the personal effects of the pandemic. Accept = acceptance of personal effects of the pandemic. Delay = perceived need to know more before acting. Refrain = perceived need to refrain from preferred action. Estimates are pooled values from 20 imputed datasets. **Bold** values represent significant correlations at $p < .05$. $^{+}$ $p = .05–.06$
