# Supplementary material for: Hidden in Plain Sight? Men's Coping Patterns and Psychological Distress Before and During the COVID-19 Pandemic
Source: Front Psychiatry. 2022 Jan 5;12:772942. doi: 10.3389/fpsyt.2021.772942 (PMC8766713; doi:10.3389/fpsyt.2021.772942)
Supplement: Supplementary file 3 [file Table_3.pdf]

Table S3. Means and CIs of Coping Strategies in LPA at T1 in Subsample that Participated at T2 During the COVID-19 Pandemic (N = 260, imputed 272)

Indicator	Relaxed Copers (C1) (*n* = 108)		Approach Copers (C2) (*n* = 121)		Dual Copers (C3) (*n* = 31)		Significant Contrasts
	M	95% CI	M	95% CI	M	95% CI	
Planning	1.76	1.58, 1.94	2.89	2.75, 3.02	2.70	2.32, 3.08	C2 & C3>C1
Active	1.87	1.74, 2.01	2.99	2.83, 3.15	2.51	2.10, 2.93	C2 & C3>C1
Positive Reframing	1.65	1.50, 1.81	2.67	2.54, 2.79	2.61	2.27, 2.96	C2 & C3>C1
Acceptance	2.01	1.83, 2.19	2.85	2.74, 2.96	2.93	2.66, 3.20	C2 & C3>C1
Humour	1.68	1.52, 1.83	2.40	2.24, 2.55	2.24	1.97, 2.51	C2 & C3>C1
Instrumental Support	1.35	1.24, 1.47	2.49	2.29, 2.69	2.35	1.92, 2.78	C2 & C3>C1
Emotional Support	1.58	1.45, 1.71	2.58	2.39, 2.77	2.41	2.00, 2.82	C2 & C3>C1
Venting	1.44	1.33, 1.55	2.01	1.89, 2.13	2.55	2.23, 2.88	C2 & C3>C1, C3>C2
Denial	1.13	1.05, 1.21	1.11	1.06, 1.16	2.52	2.24, 2.80	C3>C1 & C2
Self-distraction	1.93	1.79, 2.07	2.29	2.17, 2.40	3.05	2.65, 3.45	C2 & C3>C1, C3>C2
Behavioural Disengagement	1.35	1.23, 1.46	1.32	1.23, 1.42	2.52	2.14, 2.89	C3>C1 & C2
Substance use	1.48	1.27, 1.68	1.60	1.44, 1.76	2.54	2.20, 2.88	C3>C1 & C2
Self-blame	1.73	1.52, 1.94	2.08	1.94, 2.22	3.16	2.92, 3.39	C3>C1 & C2, C2>C1
Religion	1.09	1.04, 1.13	1.41	1.29, 1.53	1.61	1.24, 1.98	C2 & C3>C1

Note. LPA = Latent Profile Analysis. CI = confidence interval.
