# Supplementary material for: Hidden in Plain Sight? Men's Coping Patterns and Psychological Distress Before and During the COVID-19 Pandemic
Source: Front Psychiatry. 2022 Jan 5;12:772942. doi: 10.3389/fpsyt.2021.772942 (PMC8766713; doi:10.3389/fpsyt.2021.772942)
Supplement: Supplementary file 4 [file Table_4.pdf]

Table S4. Means and CIs of Coping Strategies in LPA at T2 during the COVID-19 Pandemic (N = 272)

	Relaxed Copers (C1) (*n* = 150)		Approach Copers (C2) (*n* = 86)		Dual Copers (C3) (*n* = 36)		
Indicator	M	95% CI	M	95% CI	M	95% CI	Significant Contrasts
Planning	1.64	1.39, 1.88	2.72	2.08, 3.36	2.46	2.19, 2.73	C2 & C3>C1
Active	1.81	1.58, 2.03	2.68	2.20, 3.15	2.11	1.88, 2.34	C2>C1
Positive Reframing	1.79	1.56, 2.02	2.61	2.18, 3.04	1.91	1.65, 2.17	C2>C1 & C3
Acceptance	2.65	2.42, 2.89	3.17	2.87, 3.48	2.48	2.20, 2.76	C2>C3
Humour	2.27	2.10, 2.43	2.45	2.27, 2.63	2.60	2.24, 2.96	
Instrumental Support	1.31	1.11, 1.50	2.26	1.70, 2.82	1.79	1.54, 2.04	C2 & C3>C1
Emotional Support	1.60	1.23, 1.97	2.66	2.30, 3.03	1.98	1.67, 2.28	C2>C1 & C3
Venting	1.37	1.14, 1.59	1.79	1.63, 1.96	2.33	2.11, 2.55	C2 & C3>C1, C3>C2
Denial	1.10	1.05, 1.14	1.10	1.04, 1.16	1.66	1.32, 1.99	C3>C1 & C2
Self-distraction	2.03	1.82, 2.23	2.57	2.33, 2.81	2.70	2.46, 2.94	C2 & C3>C1
Behavioural Disengagement	1.22	1.16, 1.28	1.22	1.07, 1.38	2.38	2.00, 2.76	C3>C1 & C2
Substance use	1.64	1.46, 1.83	1.76	1.44, 2.07	2.36	1.98, 2.75	C3>C1
Self-blame	1.21	1.14, 1.27	1.42	1.27, 1.56	2.74	2.26, 3.21	C3>C1 & C2, C2>C1*
Religion	1.16	1.06, 1.26	1.46	1.22, 1.70	1.26	1.06, 1.46	

Note. LPA = Latent Profile Analysis. CI = confidence interval. * Near significant contrast, CI overlaps 0.01
