# Supplementary material for: Hidden in Plain Sight? Men's Coping Patterns and Psychological Distress Before and During the COVID-19 Pandemic
Source: Front Psychiatry. 2022 Jan 5;12:772942. doi: 10.3389/fpsyt.2021.772942 (PMC8766713; doi:10.3389/fpsyt.2021.772942)
Supplement: Supplementary file 5 [file Table_5.pdf]

Table S5. Marginal Means of Cognitive Coping Appraisals of COVID-19 stressors by T1 Coping Class and Effect Sizes of Comparisons between Classes

Outcome	Relaxed Copers		Approach-Copers		Dual Copers		Approach vs Relaxed		Avoidant vs Relaxed		Avoidant vs Approach	
	M	95% CI	M	95% CI	M	95% CI	*d*	95% CI	*d*	95% CI	*d*	95% CI
Threat	2.63	2.46, 2.80	2.51	2.35, 2.67	2.59	2.25, 2.92	-0.13	-0.38, 0.12	-0.04	-0.43, 0.35	0.09	-0.30, 0.47
Harm	2.67	2.49, 2.86	2.84	2.66, 3.01	2.79	2.42, 3.15	0.17	-0.08, 0.42	0.12	-0.27, 0.51	-0.05	-0.43, 0.33
Challenge	3.04	2.88, 3.20	3.12	2.97, 3.27	3.07	2.77, 3.36	0.09	-0.16, 0.35	0.03	-0.35, 0.42	-0.06	-0.44, 0.32
Alter	2.46	2.25, 2.68	2.74	2.54, 2.93	2.83	2.43, 3.23	0.25	-0.01, 0.50	0.31	-0.08, 0.70	0.08	-0.30, 0.46
Accept	4.17	4.00, 4.34	4.28	4.13, 4.44	3.96	3.62, 4.30	0.12	-0.13, 0.37	-0.23	-0.61, 0.16	-0.34	-0.72, 0.04
Info	2.92	2.73, 3.11	3.04	2.86, 3.21	3.06	2.69, 3.42	0.12	-0.13, 0.37	0.14	-0.25, 0.52	0.02	-0.36, 0.40
Refrain	3.22	3.01, 3.43	3.18	2.98, 3.38	3.32	2.90, 3.73	-0.04	-0.29, 0.22	0.09	-0.30, 0.48	0.12	-0.26, 0.50

Note. CI = confidence interval. *d* = standardised difference between coping groups. Estimates are pooled values from 20 imputed datasets.
