# Supplementary material for: Hidden in Plain Sight? Men's Coping Patterns and Psychological Distress Before and During the COVID-19 Pandemic
Source: Front Psychiatry. 2022 Jan 5;12:772942. doi: 10.3389/fpsyt.2021.772942 (PMC8766713; doi:10.3389/fpsyt.2021.772942)
Supplement: Supplementary file 6 [file Table_6.pdf]

Table S6. Unadjusted Means and Effects for Comparisons of Psychopathology Symptoms and Coping Appraisals by Coping Classes Cross-sectionally at T1 and T2 and Longitudinally

Outcome	Relaxed Copers		Approach-Copers		Dual Copers		Approach vs Relaxed		Dual vs Relaxed		Dual vs Approach	
	M	95% CI	M	95% CI	M	95% CI	*d*	95% CI	*d*	95% CI	*d*	95% CI
Stress												
T1–T1	10.70	8.98, 12.42	12.63	10.97, 14.29	21.35	18.00, 24.71	0.20	-0.05, 0.46	**1.13**	0.72, 1.54	**0.90**	0.51, 1.30
T2–T2	11.16	9.85, 12.46	12.10	10.38, 13.82	26.51	23.85, 29.17	0.12	-0.15, 0.38	**1.89**	1.48, 2.30	**1.77**	1.32, 2.22
T1–T2	12.00	10.20, 13.80	13.76	12.10, 15.42	17.42	14.03, 20.82	0.18	-0.07, 0.44	**0.56**	0.16, 0.95	0.38*	-0.01, 0.76
Anxiety												
T1–T1	4.40	3.13, 5.67	5.54	4.36, 6.71	16.15	13.55, 18.76	0.17	-0.09, 0.42	**1.67**	1.24, 2.11	**1.53**	1.11, 1.94
T2–T2	3.58	2.61, 4.54	3.84	2.56, 5.11	13.72	11.75, 15.70	0.04	-0.22, 0.31	**1.69**	1.28, 2.09	**1.64**	1.20, 2.08
T1–T2	3.91	2.67, 5.16	4.49	3.33, 5.64	10.68	8.36, 13.01	0.09	-0.17, 0.34	**1.00**	0.60, 1.41	**0.93**	0.53, 1.32
Depression												
T1–T1	9.37	7.52, 11.22	9.00	7.22, 10.77	20.46	16.79, 24.13	-0.04	-0.29, 0.22	**1.09**	0.68, 1.50	**1.11**	0.71, 1.51
T2–T2	8.61	7.26, 9.97	9.53	7.75, 11.32	24.00	21.24, 26.76	0.11	-0.16, 0.37	**1.81**	1.40, 2.22	**1.71**	1.26, 2.15
T1–T2	10.04	8.20, 11.89	10.47	8.77, 12.18	15.81	12.32, 19.31	0.04	-0.21, 0.30	**0.58**	0.18, 0.97	**0.54**	0.15, 0.92
Anger												
T1–T1	27.24	25.52, 28.97	29.07	27.48, 30.66	38.89	35.57, 42.21	-0.06	-0.06, 0.45	**1.24**	0.82, 1.65	**1.06**	0.66, 1.46
T2–T2	20.98	19.90, 22.06	20.91	19.48, 22.33	35.47	33.27, 37.68	-0.28	-0.28, 0.25	**2.15**	1.72, 2.57	**2.16**	1.68, 2.64
T1–T2	22.05	20.53, 23.58	22.13	20.71, 23.55	28.57	25.61, 31.53	-0.24	-0.24, 0.26	**0.78**	0.38, 1.18	**0.78**	0.38, 1.17

Note. CI = confidence interval. *d* = standardised difference between coping groups. T1–T1 = coping and psychopathology at timepoint one. T2–T2 = psychopathology or appraisal at timepoint two. T1–T2 = T2 psychopathology regressed on T1 coping profile. Estimates are pooled values from 20 imputed datasets. **Bold** values are significant at $p<.05$. *bordering significance; $p = .058$.
