# Supplementary material for: Hidden in Plain Sight? Men's Coping Patterns and Psychological Distress Before and During the COVID-19 Pandemic
Source: Front Psychiatry. 2022 Jan 5;12:772942. doi: 10.3389/fpsyt.2021.772942 (PMC8766713; doi:10.3389/fpsyt.2021.772942)
Supplement: Supplementary file 7 [file Table_7.pdf]

Table S7. Unadjusted and Adjusted Means and CI for Psychopathology Symptoms and Coping Appraisals During the COVID-19 Pandemic for Each Longitudinal Coping Pattern

Outcome	Stable RA Copers		New RA Copers		Stable Dual Copers		New Dual Copers	
	M	95% CI	M	95% CI	M	95% CI	M	95% CI
Psychopathology								
Stress	11.51	10.41, 12.60	11.41	7.63, 15.20	26.70	22.23, 31.17	26.40	23.07, 29.73
Stress[Adj]	12.37	11.36, 13.37	8.44	4.76, 12.11	21.63	17.29, 25.98	23.74	20.67, 26.82
Anxiety	3.42	2.62, 4.21	6.40	3.70, 9.09	17.29	14.01, 20.57	11.73	9.30, 14.16
Anxiety[Adj]	4.12	3.37, 4.87	3.51	0.82, 6.21	12.02	8.71, 15.32	10.57	8.29, 12.84
Depression	8.92	7.79, 10.06	9.22	5.31, 13.14	25.99	21.35, 30.63	22.89	19.44, 26.35
Depression[Adj]	9.82	8.79, 10.85	6.79	3.28, 10.29	18.95	14.38, 23.51	20.56	17.40, 23.72
Anger	20.85	19.95, 21.74	22.09	19.06, 25.13	38.57	34.89, 42.26	33.74	30.98, 36.49
Anger[Adj]	21.27	20.40, 22.14	20.42	17.42, 23.41	34.70	30.82, 38.58	33.40	30.75, 36.05
Appraisals of COVID-19 stressors								
Threat	2.52	2.40, 2.64	2.30	1.86, 2.74	3.03	2.55, 3.51	2.97	2.62, 3.33
Harm	2.70	2.57, 2.82	2.33	1.84, 2.82	3.49	2.98, 4.01	3.32	2.94, 3.70
Challenge	3.13	3.03, 3.24	3.45	3.06, 3.83	2.48	2.03, 2.93	2.60	2.62, 2.93
Appraisals of coping options								
Alter	2.64	2.49, 2.79	3.22	2.69, 3.74	2.24	1.63, 2.85	2.30	1.84, 2.76
More Info	2.92	2.79, 3.05	2.78	2.31, 3.24	3.49	2.94, 4.04	3.55	3.15, 3.95
Refrain	3.16	3.01, 3.30	2.96	2.41, 3.51	3.87	3.28, 4.46	3.60	3.16, 4.04
Accept	4.23	4.12, 4.35	4.11	3.65, 4.57	3.73	3.25, 4.21	4.20	3.84, 4.55

Note. CI = confidence interval. Stable RA = Relaxed/Approach Copers at T1 & T2. New RA Copers = Relaxed/Approach Copers at T2 who were previously Dual Copers at T1. Stable Dual Copers = Dual Copers at T1 & T2. New Dual Copers = Dual Copers at T2 who were previously Relaxed/Approach Copers at T1. [Adj] = main effects model adjusted for potential confounders. Estimates are pooled values from 20 imputed datasets.
