# Supplementary material for: Hidden in Plain Sight? Men's Coping Patterns and Psychological Distress Before and During the COVID-19 Pandemic
Source: Front Psychiatry. 2022 Jan 5;12:772942. doi: 10.3389/fpsyt.2021.772942 (PMC8766713; doi:10.3389/fpsyt.2021.772942)
Supplement: Supplementary file 8 [file Table_8.pdf]

Table S8. Effect Sizes for Differences in Coping Appraisals and Psychopathology Symptoms During the COVID-19 Pandemic Between Longitudinal Coping Patterns

	New RA vs Stable RA		Stable Dual vs Stable RA		New Dual vs Stable RA		Stable Dual vs New RA		New Dual vs New RA		New Dual vs Stable Dual	
Outcome	*d*	95% CI	*d*	95% CI	*d*	95% CI	*d*	95% CI	*d*	95% CI	*d*	95% CI
Psychopathology												
Stress	-0.01	-0.47, 0.45	**1.86**	1.27, 2.44	**1.82**	1.36, 2.28	**1.80**	0.96, 2.62	**1.79**	1.07, 2.49	-0.04	-0.72, 0.64
Stress^{Adj}	**-0.52**	-0.98, -0.06	**1.23**	0.66, 1.80	**1.52**	1.06, 1.97	**1.60**	0.79, 2.40	**1.93**	1.19, 2.65	0.27	-0.41, 0.96
Anxiety	**0.50**	0.04, 0.96	**2.34**	1.74, 2.94	**1.40**	0.95, 1.85	**1.79**	0.95, 2.60	**0.88**	0.25, 1.51	**-0.93**[a]	-1.64, -0.21
Anxiety^{Adj}	-0.11	-0.57, 0.35	**1.40**	0.82, 1.97	**1.15**	0.70, 1.59	**1.39**	0.60, 2.16	**1.21**	0.55, 1.86	-0.25	-0.93, 0.43
Depression	0.03	-0.42, 0.49	**2.00**	1.41, 2.58	**1.64**	1.18, 2.09	**1.91**	1.05, 2.74	**1.57**	0.88, 2.25	-0.37	-1.05, 0.32
Depression^{Adj}	-0.39	-0.85, 0.07	**1.18**	0.61, 1.75	**1.39**	0.94, 1.84	**1.49**	0.69, 2.27	**1.75**	1.04, 2.45	0.20	-0.48, 0.88
Anger	0.19	-0.27, 0.64	**2.65**	2.04, 3.26	**1.93**	1.46, 2.39	**2.40**	1.47, 3.30	**1.71**	1.00, 2.40	**-0.72**[a]	-1.41, -0.01
Anger^{Adj}	-0.13	-0.59, 0.33	**2.05**	1.46, 2.64	**1.86**	1.40, 2.32	**2.06**	1.18, 2.91	**1.95**	1.21, 2.68	-0.19	-0.87, 0.49
Stressor Appraisals												
Threat	-0.24	-0.70, 0.22	**0.57**[b]	0.005, 1.13	**0.50**	0.07, 0.93	**0.76**[b]	0.03, 1.48	**0.71**	0.09, 1.33	-0.07	-0.75, 0.61
Harm	-0.40	-0.86, 0.06	**0.87**	0.31, 1.44	**0.69**	0.25, 1.12	**1.10**	0.34, 1.84	**0.97**	0.33, 1.60	-0.18	-0.86, 0.50
Challenge	0.39	-0.07, 0.85	**-0.79**	-1.35, -0.22	**-0.64**	-1.08, -0.21	**-1.14**	-1.88, -0.38	**-1.02**	-1.65, -0.38	0.15	-0.53, 0.83
Options Appraisals												
Alter	**0.51**	0.05, 0.97	-0.36	-0.92, 0.21	-0.30	-0.73, 0.13	**-0.84**	-1.57, -0.11	**-0.80**	-1.42, -0.17	0.05	-0.63, 0.73
Info	-0.14	-0.60, 0.32	**0.58**	0.02, 1.15	**0.65**	0.21, 1.08	0.69[+]	-0.04, 1.40	**0.76**	0.14, 1.38	0.06	-0.62, 0.74
Refrain	-0.19	-0.65, 0.27	**0.68**	0.11, 1.24	0.42[#]	-0.01, 0.85	**0.76**	0.03, 1.48	0.55	-0.06, 1.16	-0.25	-0.93, 0.43
Accept	-0.13	-0.59, 0.33	**-0.56***	-1.12, 0.01	-0.03	-0.46, 0.40	-0.38	-1.09, 0.32	0.09	-0.51, 0.69	0.54	-0.15, 1.23

Note. CI = 95% confidence interval. *d* = standardised difference between coping groups. ^{Adj} model adjusted for potential confounders. [a] Removal of influential case(s) reduced the magnitude of these effects and widened confidence intervals: anxiety *d* = -0.46, 95% CI [-1.19, 0.28], anger *d* = -0.46, 95% CI [-1.16, 0.25]. [b] Use of robust regressions to manage potential heteroskedasticity did not meaningfully alter the magnitude of these effect but widened confidence intervals: Stable Dual vs Stable RA *d* = 0.56, 95 CI [-0.01, 1.12], Stable Dual vs New RA *d* = 0.71, 95% CI [-0.02, 1.43]). **Bold** effects are significant at $p<.05$. * Pairwise contrast of marginal means $p <.05$. [+] Pairwise contrast of marginal means $p = .05$. [#] Pairwise contrast of marginal means $p = .06$.
